# Beyond the Coagulation Cascade: Vitamin K and Its Multifaceted Impact on Human and Domesticated Animal Health

**DOI:** 10.3390/cimb46070418

**Published:** 2024-07-04

**Authors:** Rebecka A. Sadler, Anna K. Shoveller, Umesh K. Shandilya, Armen Charchoglyan, Lauraine Wagter-Lesperance, Byram W. Bridle, Bonnie A. Mallard, Niel A. Karrow

**Affiliations:** 1 Department of Animal Biosciences, University of Guelph, Guelph, ON N1G 2W1, Canada; sadlerr@uoguelph.ca (R.A.S.); ashovell@uoguelph.ca (A.K.S.); ushand@uoguelph.ca (U.K.S.); 2 ImmunoCeutica Inc., Cambridge, ON N1T 1N6, Canada; armencharch@protonmail.com (A.C.); lwagterl@uoguelph.ca (L.W.-L.); bbridle@uoguelph.ca (B.W.B.); bmallard@ovc.uoguelph.ca (B.A.M.); 3 Advanced Analysis Centre, University of Guelph, Guelph, ON N1G 2W1, Canada; 4 Department of Pathobiology, University of Guelph, Guelph, ON N1G 2W1, Canada

**Keywords:** vitamin K, phylloquinone, MK-7, MK-4, coagulation, anti-inflammation, immunomodulation, neuroprotection, microbiota, apoptosis

## Abstract

Vitamin K (VK) is an essential micronutrient impacting many systems in the body. This lipid-soluble vitamin is found in various plant and animal products and is absorbed via the lymphatic system. This biomolecule’s importance to human health includes but is not limited to its promotion of brain, cardiovascular, bone, and immune functions. These biological properties are also necessary for maintaining domesticated animal health. The synergistic impact of both VK and vitamin D (VD) maximizes these health benefits, specifically for the circulatory and skeletal systems. This manuscript reviews VK’s properties, molecular structures, nutrikinetics, mechanisms of action, daily requirements, safety in supplemental form, biomarkers used for its detection, and impacts on various organs. The purpose of synthesizing this information is to evaluate the potential uses of VK for the treatment or prevention of diseases.

## 1. Introduction 

Vitamin K (VK) was discovered in 1935 as a fat-soluble, antihemorrhagic factor in chickens [1]. While VK is primarily known for enabling normal blood coagulation, this biomolecule also plays critical roles in improving bone health, preventing vascular calcification, enhancing brain function, modulating immune system function, attenuating inflammation, lowering cancer incidence, and much more [2]. Therefore, the purpose of this paper is to highlight the multifaceted impacts of VK on human and animal health. By analyzing various studies, the known molecular mechanisms behind VK’s actions are also summarized and brought up to date.

## 2. Vitamin K Types, Sources, and Bioavailability

**Figure 1:**
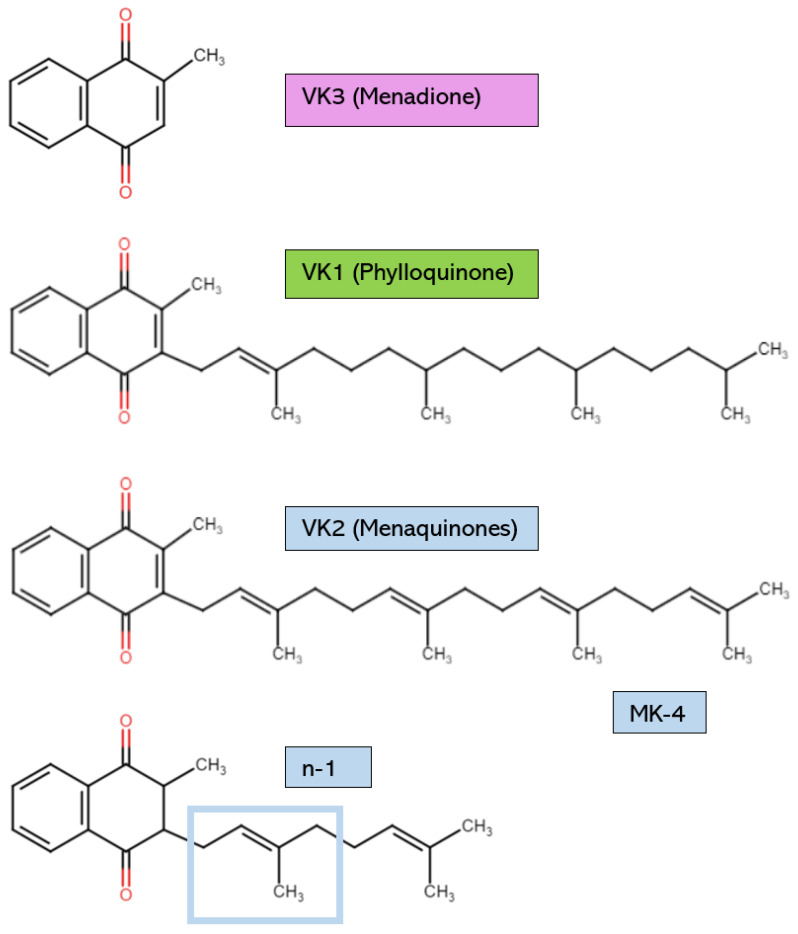
Vitamin K structures and descriptions. MK-4: menaquinone-4. The structure of any MK can be created by using the structural formula n-1, where n is the number of unsaturated β-isoprenoid units [MK-n].

The various forms of VK possess structural distinctions, as shown in Figure 1. However, all have the same core 2-methyl-1,4-napthoquinone ring [2]. VK1, or phylloquinone, is synthesized by cyanobacteria and is found in green plants and algae. VK1 can be distinguished by its long phytyl side chain [3]. Plants rich in VK1 include broccoli, dandelion leaves, spinach, cabbage, avocado, kiwi, and stinging nettles [4,5]. Leafy greens have the highest amount of VK1 [5]. However, dietary VK1 sources have low bioavailability due to the binding of phylloquinone to intracellular plant structures like chloroplasts [4]. Some estimate that the VK1 found in vegetables is only absorbed at a rate of 5–10% [6]. Therefore, it is necessary to eat saturated fats alongside dietary VK sources or to consume purified VK1 from commercial sources to increase absorption [4]. For example, the phylloquinone in plant oils is absorbed easily due to the presence of fats, which increase VK’s bioavailability [7]. Generally, the VK1 found in supplements is chemically synthesized, not extracted from natural plant sources [8,9]. However, from chemical synthesis and natural extraction, the yield of VK1 is low due to the complicated pretreatment procedures and the numerous reaction steps [10].

The other significant form of VK is VK2, which possesses an unsaturated isoprenoid side chain. VK2 is found as a hydrophobic long-chain menaquinone (MK) [9]. Different MK subtypes exist, from MK-4 to MK-13, according on the number of unsaturated β-isoprenoid units [4,11]. Certain aerobic and anaerobic bacteria biosynthesize VK2 to use as electron carriers in redox reactions within bacterial cytoplasmic membranes [4,12]. The bacterial species that are known to produce VK2 as a result of their metabolic activities include *Escherichia coli*, *Bacillus cereus*, *Mycobacterium tuberculosis*, *Staphylococcus aureus*, *Sarina lutea*, and many others [13]. Examples of VK2 dietary sources include cheese, sauerkraut, chicken, and beef products [2]. Natto is a fermented soy product that is high in MK-7; for comparison, natto contains 100 times the amount of MK-7 found in cheese [14]. The production of this Japanese food requires *Bacillus subtilis*, which ferments the soybeans after they have been boiled or steamed [14]. MK-7 is released as a microbial metabolite. In supplements, VK2 is typically in the form of MK-4 or MK-7. MK-7 is commonly extracted from natto, whereas MK-4 is usually organically synthesized by other bacteria [9]. Even though all VK2 subtypes can be chemically synthesized to form a supplemental product with high purity and biological activity, microbial fermentation is now considered a more biologically safe, economically efficient, and environmentally friendly way of producing VK2 [10]. 

VK3, also known as menadione (MK-0), is typically a synthetic form of VK and has no isoprenoid side chain. VK3 is not used often in supplements due to reports of some adverse events [15]. It is also not a bioactive cofactor for the VK-dependent carboxylase enzyme known as γ-glutamylcarboxylase (GGCX) [16]. Therefore, VK3 is not covered extensively in this review. However, VK3 can elicit antimicrobial actions against multi-drug-resistant bacteria, so VK3 may soon be applied in human medicine as research continues [17,18,19,20]. Finally, VK4, VK5, VK6, and VK7 can be synthetically derived, but they have not been the focus of VK research [15]. 

Though all VK2 forms share some common mechanisms of action, they are unique in their biosynthetic origins and functional effectiveness. For example, while most MKs are synthesized by different strains of bacteria, MK-4 is produced from other VK subtypes in the endoplasmic reticulum in humans by the enzyme UbiA prenyltransferase containing 1 encoded by the gene *UBIAD1*, which appears to be expressed to varying degrees in many tissues [21,22]. Initially, an unknown enzyme catalyzes the removal of the VK side chain to form a transient intermediate metabolite of VK3 [21,22]. Next, the enzyme encoded by *UBIAD1* adds a geranylgeranyl side chain to make MK-4 [23]. These reactions are presumed to happen chiefly in extrahepatic tissues such as the kidney, pancreas, salivary glands, testes, visceral fat, and brain, where MK-4 accumulates [23]. VK1 cannot change into MK-4 in the liver, and MK-4 does not remain in either the liver or serum for very long [24]. Intestinal bacteria are also not responsible for endogenous MK-4 production [25]. This was confirmed in a study showing that MK-4 can be synthesized in germ-free rats [25]. However, bacteria in the large intestine synthesize longer-chained MKs, but their bioavailability and bioactivity are very low [24,26]. Examples of MK-producing commensal bacteria in the human gut microbiome include *Eubacterium lentum*, *Veillonella*, *Enterobacteria*, and *Bacteroides* [10,27]. In rats, bacterially synthesized VK2 may be absorbed passively in the colon to satisfy VK requirements [28]. Whether this is true for humans remains to be determined. Therefore, it is presumed that people must primarily obtain VK from their diet instead of relying on VK synthesis by colonic bacteria [29]. 

There are also notable variations in the physiological half-lives of different VK forms. In people, the physiological half-lives of VK1 and MK-7 are 1–2 h and 3 days, respectively [4]. After 24 h, VK1 and MK-4 disappear entirely from the circulatory system [24]. However, supplementation with MK-7 substantially increases serum VK concentrations over multiple days compared to MK-4 and VK1, highlighting MK-7’s superior ability to be used and persist in the body [9,30]. Overall, not all VK forms are equal in their absorption and distribution. Therefore, the varying physiological half-lives of the VK forms in the serum should be considered when determining VK supplementation strategies.

There are also varying potencies and bioactivities of different VK forms that impact their efficacy as a GGCX cofactor. First, the potency of MK-7 is three to four times higher than that of VK1 on a molar basis [31]. Research also indicates that MK-7 elicits the same beneficial biological effects as MK-4 and VK1 at a much lower dose, highlighting its superior bioactivity [11]. These may be the reasons why MK-7 plays a more significant role than VK1 in both hepatic and extrahepatic protein carboxylation [9]. For example, the administration of MK-7 over 40 days resulted in a higher ratio of carboxylated to undercarboxylated osteocalcin, a gamma (γ)-carboxyglutamic acid (Gla) protein needing VK for its carboxylation, than supplementing with VK1 [9]. Compared to VK1, MK-4 fixes more carbon dioxide as a cofactor in the GGCX enzyme at a lower concentration; MK-4 also achieves a half-maximal reaction velocity at a concentration three times lower than VK1 [16]. Therefore, MK-7 appears to have the highest potency and bioactivity, followed by MK-4 and VK1. Overall, it is necessary to recognize the varying biological efficacies of different VK forms when deciding on the best supplemental or dietary VK source to consume. However, current dietary recommendations do not take this into consideration.

## 3. Vitamin K Nutrikinetics 

**Figure 2:**
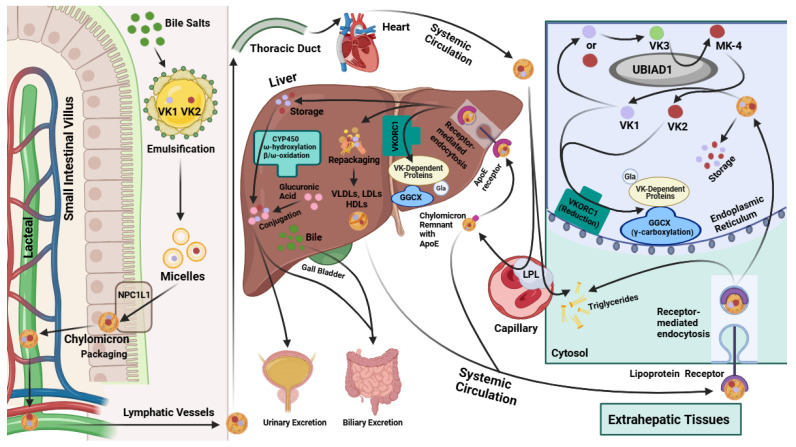
Vitamin K absorption, transport and metabolism in hepatic and extrahepatic tissues. VK: vitamin K. GGCX: γ-glutamyl carboxylase. VKORC1: vitamin K epoxide reductase complex subunit 1. CYP450: cytochrome P450. VLDLs: very-low-density lipoproteins. LDLs: low-density lipoproteins. HDLs: high-density lipoproteins. LPL: lipoprotein lipase. Gla: gamma (γ)-carboxyglutamic acid. MK-4: menaquinone-4 (subtype of Vitamin K2). NPC1L1: Niemann-Pick C1-like 1.

The knowledge of VK absorption is primarily based on research involving VK1 [11]. Further research is needed to confirm the precise mechanism of VK2 uptake and whether it differs from that of VK1. However, the current scientific evidence suggests that the absorption of dietary VK occurs in the small intestine after it is emulsified by bile salts alongside triglycerides (TGs) [32,33]. As shown in Figure 2, this produces micelles carrying VK that enter gastrointestinal enterocytes though Niemann-Pick C1-like 1 (NPC1L1) transporters and are packaged into chylomicrons [32,33]. These chylomicrons then flow into the villi’s lymph-carrying lacteals to the thoracic duct and into the systemic circulation [32]. VK is primarily transported around the body via the triacylglycerol-rich lipoprotein fraction of the blood [34]. 

While passing through capillaries, the TGs in the chylomicrons are metabolized by extracellular lipoprotein lipase (LPL). The emptied chylomicrons, now referred to as chylomicron remnants, still contain lipophilic VK in their core [32]. These chylomicron remnants express ApoE on their surface, a 34 kDa lipoprotein that binds to various lipoprotein receptors expressed in different tissues [7]. Notably, some of the chylomicron remnants are endocytosed by hepatocytes via binding to ApoE receptors [32,34] (Figure 2). Other chylomicron remnants are circulated systemically and taken up by extrahepatic tissues, like the muscle and bone, through lipoprotein-receptor-mediated endocytosis [32,35] (Figure 2). 

Due to their increased hydrophobicity, the longer-chain MKs, like MK-7 and MK-9, are usually repackaged into lipoproteins in the liver and transported systemically [4,9,32,36]. The uptake of circulating lipoproteins containing these MKs is facilitated mainly through receptor-mediated endocytosis in various target tissues [32]. VK2 primarily resides in extrahepatic tissues, including the bones, testes, pancreas, kidneys, lungs, and blood vessel walls [34]. However, the longer-chained MKs can also be stored in the liver and have a slower turnover rate than VK1 due to their high affinity for hepatic membranes [31]. In the liver, VK2 types such as MK-7 play a significant role in the carboxylation of hepatic Gla proteins required for blood coagulation [31,32] (Figure 2). Studies suggest that MK-7 is first used for hepatic Gla protein carboxylation, and, afterward, the remaining MK-7 is distributed for extrahepatic protein carboxylation [31]. Likewise, VK1 is also first used to carboxylate hepatic proteins, temporarily accumulating in the liver, before being distributed to other bodily tissues for extrahepatic Gla protein carboxylation [34,36].

The hepatic metabolism of excess VK1 and VK2 is carried out by various cytochrome P450 enzymes, including those encoded by the genes *CYP4F2*, *CYP4A,* and different *CYP3A* isoforms [31,32] (Figure 2). To increase the rate of VK metabolism, VK binding to hepatic steroid and xenobiotic receptor (SXR) upregulates the expression of *CYP4A* isoforms [32]. Notably, MK-4 binds with a higher affinity than VK1 to the pregnane X receptor (PXR), which is homologous to SXR [37]. This cytochrome-P450-mediated metabolism of VK occurs through a series of reactions involving ω oxidation, ω hydroxylation, and β oxidation [31,32,37], which ultimately lead to VK being metabolized into two carboxylic acid aglycone metabolites, a five-carbon (5C; 2-methyl-3-(3′-3′-carboxymethylpropyl)-1,4-napthoquinone) and a seven-carbon (7C; 2-methyl-3-(5′-carboxy-3′-methyl-2′-pentenyl)-1,4-naphthoquinone) side-chain aglycone [32,38]. After conjugation to glucuronic acid (Figure 2), these VK metabolites are excreted through the biliary and urinary tracts in the feces and urine, respectively [31,32]. 

## 4. Mechanisms of Action

### 4.1. Cofactor for GGCX

**Figure 3:**
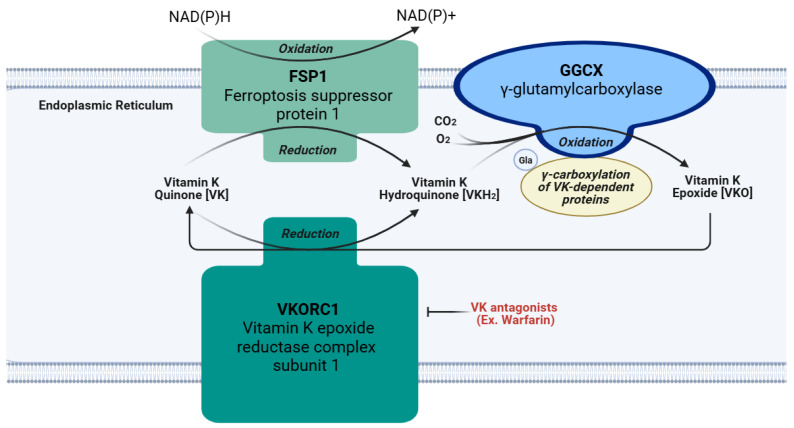
Vitamin K cycle and action of pharmaceutical antagonists. VK: vitamin K quinone. VKH_2_: hydroquinone. VKO: vitamin K epoxide. FSP1: ferroptosis suppressor protein 1. GGCX: γ-glutamyl carboxylase. VKORC1: vitamin K epoxide reductase complex subunit 1. CO_2_: carbon dioxide. O_2_: oxygen.

The chief function of VK is an essential cofactor for GGCX, as depicted in Figure 3 [5]. This enzyme is responsible for the γ-glutamyl carboxylation of peptide glutamate residues, which converts them into Gla residues [4,5]. The first step in this process requires the reduction of VK from quinone to hydroquinone (VKH_2_) by the VKORC1 enzyme located on the luminal membrane of the rough endoplasmic reticulum [4]. The nicotinamide adenine dinucleotide (phosphate)-dependent oxidoreductase, known as ferroptosis suppressor protein 1 (FSP1), is another enzyme frequently embedded in the endoplasmic reticulum that converts VK quinone to hydroquinone, especially when VKORC1 is inhibited by a VK antagonist drug like warfarin [39,40]. Next, GGCX deprotonates VKH_2_, putatively forming an alkoxide by adding oxygen; this base is thought to induce the formation of a carbanion at the γ location on a glutamic acid residue of Gla proproteins. Subsequently, carbon dioxide is presumed to react with the carbanion causing γ-glutamyl carboxylation [4]. After this, the oxidized hydroquinone, referred to as VK epoxide, is reduced by VKORC1 to form hydroquinone again, and this cycle repeats itself for all the necessary carboxylation reactions. Once all the necessary γ-glutamyl carboxylation reactions are completed on a Gla proprotein in the endoplasmic reticulum, the Gla proprotein usually continues through the secretory pathway into the Golgi apparatus for the removal of its prosequence and secretion [4]. Since some Gla proteins lack this prosequence, or can be excreted in an undercarboxylated state, γ-glutamyl carboxylation may not be necessary for all Gla proteins [4]. Although these Gla proteins perform many functions, one shared feature is their ability to bind calcium [41]. Binding to calcium alters the Gla protein’s structure and optimizes its biological activity [39]. Therefore, activating Gla proteins is the primary role of VK in the body. A list of the Gla proteins covered in this review and their functions are included in Table 1.

### 4.2. Antioxidant Functions

Another serendipitously discovered function of VK is its role in preventing oxidative damage. Oxidative cell death, resulting from various causes, leads to the accumulation of reactive oxygen species (ROS) and the lowering of glutathione (GSH) levels, a necessary antioxidant [42]. Apart from their function as cofactors for GGCX, VK1 and MK-4 have demonstrated usefulness in preventing cellular death by inhibiting the buildup of cytotoxic ROS in immature cortical neurons and primary oligodendrocytes [42]. The mechanism of action through which VK accomplishes this has been attributed to its inhibition of 12-lipoxygenase (12-LOX) activation, which is partly responsible for the metabolism of arachidonic acid released from membrane phospholipids [43]. Without VK1 and MK-4 inhibiting 12-LOX, free radicals and peroxides would accumulate, causing oxidative damage to the cell [43]. Therefore, reducing oxidative stress may be one potential therapeutic use for VK that warrants further investigation.

### 4.3. Antiferroptotic Activity

Recent research has shed light on VK’s radical trapping and antioxidant properties within the context of ferroptosis [44,45]. Ferroptosis is a type of cell death involving iron-dependent lipid peroxidation. It is linked to several diseases, including cancers, certain types of cardiomyopathy, cystic fibrosis, hemochromatosis, Huntington’s disease, and Parkinson’s disease [46]. Various forms of VK, including VK1, MK-4, and VK3, possess antiferroptotic properties based on studies involving mouse-cultured fibroblasts, human cancer cells, fibrosarcomas, pancreatic cells, and neurons [44,47]. Selenium-dependent GSH peroxidase-4 (Gpx4) is the principal ferroptosis regulator, which is achieved through its involvement in redox reactions that combat lipid peroxidation [47]. To test the efficacy of potential antiferroptotic compounds, *GPX4* knockout (KO) mice received MK-4 supplementation before an ischemia–reperfusion challenge. In comparison to the control group, the VK treatment effectively decreased injuries associated with ischemia–reperfusion in the kidney and liver [47]. The pathological consequences of ferroptosis attenuated by MK-4 treatment included lipid peroxidation, cellular death, and inflammation in the liver, as well as renal tissue damage and poor function. Thus, Mishima et al. concluded that the VK treatments effectively reduced ferroptosis in vitro and in vivo. VK’s antioxidant mechanism of action partly involves the production of VKH_2_, catalyzed from VK quinone by FSP1 [47,48]. This critical VK reduction pathway bypasses the canonical VK cycle using VKORC1 [49]. Overall, VK is a valuable antioxidant whose reduction of cellular ferroptosis may be used in upcoming medical applications.

### 4.4. Anti-Inflammatory Roles

Additionally, within the context of inflammatory processes, VK2 has demonstrated many unique mechanisms of action. In the first case, VK1, MK-4, and VK3 inhibit the release of the interleukin (IL)-6 cytokine from lipopolysaccharide (LPS)-activated gingival fibroblasts, although the mechanism was unknown [50]. These authors found VK3 was most effective, followed by MK-4, then VK1. In recent research, MK-4 suppressed LPS-induced expression of the genes encoding various proinflammatory cytokines, such as IL-6, tumor necrosis factor (TNF)-α, and IL-1β, in macrophage-like cells; the use of warfarin demonstrated VK’s anti-inflammatory effects apart from its function as a GGCX cofactor [51]. These researchers found that this effect was due to the ability of MK-4 to significantly limit the phosphorylation of inhibitor of nuclear factor-kappa B (IκB) kinase α/β, an action that reduced the downstream phosphorylation of I*κ*B and p65, as well as the nuclear translocation of nuclear factor (NF)-κB [51]. Thus, this process impairs the ability of the proinflammatory transcription factor NF-κB to elicit the expression of inflammatory cytokines. These anti-inflammatory effects may partially explain why high levels of VK can also be detected in extrahepatic tissues, but more research is needed to determine the exact process through which VK reduces inflammation [51]. 

### 4.5. Ligand for SXR/PXR

Another recently discovered mechanism of action of VK, reviewed by Azuma et al., 2014, is its function as a ligand for SXR/PXR, encoded by the *NR1I2* gene. Being expressed primarily in the intestine and liver, the ligands for SXR/PXR include VK, secondary bile acids, and xenobiotic compounds, including some pharmaceutical drugs. Activated SXR/PXR functions as a transcription factor after heterodimerizing with the 9-cis retinoid acid receptor (RXR), and its target is the SXR-responsive element (SXRE), located in enhancer or promotor regions of the corresponding genes. The proteins subsequently synthesized after SXRE engagement typically have functions related to drug metabolism, detoxification, and elimination; examples include enzymes in the cytochrome P450 family such as those encoded by the genes *CYP3A4* and *CYP2C8* [52]. However, SXR/PXR has also been found in osteoblast cell lines, blood mononuclear cells, kidney tissue, and lung tissue, indicating that VK may elicit relevant physiological effects through this mechanism throughout the body [53,54]. 

### 4.6. Activation of Other Biological Targets

Researchers have found that VK activates several other biological targets as well. For example, MK-4 can induce the expression of the genes encoding growth differentiation factor 15 (GDF15) and stanniocalcin 2 (STC2), in addition to its GGCX or SXR-binding activities [55]. GDF15 is a significant protein that affects cellular proliferation, adhesion, and growth, while STC2 is a glycoprotein hormone [55]. The MK-4-induced expression of these proteins is linked partially to its ability to elicit protein kinase A (PKA) activity [56]. Another study found a similar effect of VK1 and VK2 activating PKA and mitogen-activated protein kinases (MAPKs) to promote neurite outgrowth [57]. Interestingly, VK2 also binds to 17β hydroxy-steroid dehydrogenase 4, an enzyme responsible for the conversion of the active estradiol (E2) to inactive estrone (E1) [58]. These authors demonstrated in vitro that VK2 decreased the ratio of E2:E1, which resulted in fewer estrogen receptor α interactions with E2 response elements and less transcription of E2 target genes. Overall, these studies demonstrate the wide range of biological targets and signaling cascades impacted by VK with potentially many more to be discovered. 

### 4.7. Roles in Cellular Death

VK can also induce apoptosis and nonapoptotic cellular death in particular cancer cells. For example, VK2 can trigger the apoptosis of leukemia cells positioning it as a potential candidate for a treatment [59]. Though the exact mechanism remains uncertain, researchers have proposed that the action is facilitated by the unsaturated side chain of VK2, which is different from the phytol side chain of VK1 [59,60]. Other researchers found that VK2 caused the apoptosis of pancreatic and ovarian cancer cells via an unknown process involving de novo protein synthesis [61]. However, these authors demonstrated that VK2 did not have this same apoptotic effect on other tumor cell lines, including stomach, colon adenocarcinoma, and liver cancer cells. A recent study reported that VK2 could elicit autophagy and nonapoptotic cellular death of specific breast cancer cell lines [62]. These researchers proposed that VK enhanced the synthesis of the endoplasmic reticulum stress-related transcription factor C/EBP homologous protein, which is a proapoptotic protein. However, the death of all the cancerous cell lines could not be attributed to this mechanism, since VK-induced ROS production leading to autophagy was also credited for causing cellular death [62]. Earlier, Karasawa et al. determined another way through which VK induced the mitochondrial-mediated apoptosis of human promyelocytic cells [63]. These authors demonstrated that the binding of a VK2 2,3-epoxide metabolite to a cysteine-166 residue on B-cell lymphoma 2 (Bcl-2) antagonist killer 1, a proapoptotic protein, elicited the secretion of cytochrome C from the mitochondria to initiate apoptosis; they also confirmed the ability of VK2 to generate ROS in these cancer cells [63]. A later study demonstrated the activation of p38 and c-Jun N-terminal kinase (JNK) MAPK by VK2, due to its ROS-generating effects, leading to the mitochondrial-induced apoptosis of human bladder cancer cells [64]. Therefore, VK appears to have many different biological mechanisms for triggering cancer cell death. An in-depth review of VK’s complex impacts on the treatment and pathogenesis of cancer has been recently conducted elsewhere [65,66,67].

## 5. Vitamin K Requirements 

The VK recommendations by the American Institute of Medicine to prevent deficiency are in Table 2 [68]. For infants, determining adequate intake levels requires multiplying the average concentration of VK1 in breast milk by the total amount consumed per day [68]. Various studies have determined that the average concentration of VK1 in breast milk is 2.5 µg/L [68]. Therefore, the Institute of Medicine recommends that 2.0 µg/day of VK1 be provided for 0–6-month-old infants and 2.5 µg/day for 7–12-month-old infants. For children, adequate intake values are crudely determined based on data from the Third National Health and Nutrition Examination Survey. In this survey, the highest median consumption of VK1 by healthy children in each age category was used to calculate the estimated average requirements (Table 2). For adults, estimated VK requirements have also been determined from the reported average consumption by healthy individuals. For men over 19 years of age, the recommended adequate intake is 120 μg/day; for women over 19 years of age, the recommended adequate intake is 90 μg/day; and the recommendation is the same for pregnant and lactating women (Table 2) [68].

It is important to note that these are only recommendations because there are insufficient data regarding what constitutes sufficient VK status. The varying bioactivities of different forms and subtypes of VK were also not considered in their recommendations. The current clinical definition of a VK deficiency is excessive bleeding due to a lack of activated clotting factors [69]. Specifically, this includes a prolonged prothrombin time and probable subsequent hemorrhaging [68]. However, since VK activates many Gla proteins, subclinical VK deficiencies could manifest as the pathological calcification of bone and cartilage when the relevant Gla proteins, like osteocalcin, are inactive [69]. In one study, subclinical VK deficiency was defined as having a plasma VK1 concentration of ≤ 0.5 nM; this was because a study conducted in 1989 considered the standard normal range of plasma VK1 concentration to be 0.5–2.5 nM [69]. However, more research is needed to determine how to identify subclinical VK deficiencies accurately and consistently using verifiable biomarkers. In this way, negative health consequences can be avoided by prophylactically administering VK supplements to those at risk.

One notable aspect of VK recommendations is that there is no upper limit set for VK daily intake since there are not enough data to determine this [68]. No harmful dose of VK has been established, and toxicity is reported rarely in both animals and humans [4]. To support the safety of VK supplementation, many studies have been carried out using high supplement doses without any reported adverse events. In one study, 107 women within the age range of 55–65 years consumed 180 μg/day of MK-7 supplement for three years, and no adverse side effects were reported by the participants [70]. In another study, 20–55-year-old male and female participants were offered 200 μg/day of MK-7 for 12 weeks [71]. Again, none of the 30 volunteers reported any adverse side effects [71]. In a different study, none of the 18–45-year-old participants taking 360 μg of MK-7 per day for 12 weeks dropped out of the study or showed any adverse effects concerning their ability to generate thrombin [36]. Another study using the same supplement concentration and duration reported no adverse events [72]. Daily consumption of natto containing 387.5 µg, 649 µg, or 882.5 µg of MK-7 for 7 days led to an increase in serum MK-7 concentrations from below 20 ng/mL to between approximately 20 ng/mL and 50 ng/mL, depending on the daily dose [73]. No side effects were reported in this study, supporting the safety of consuming relatively high levels of MK-7 for a short time. A study supplementing rats with 20 mg/kg of MK-7 did not elicit any toxic effects [15]. Because of the widespread consumption of natto in Japan, the MK-7 plasma levels of the Japanese are typically much higher than those of European populations. For example, a Japanese study recorded plasma MK-7 levels at 6.99 ng/mL [74], contrasting an Italian study that recorded median MK-7 levels in healthy controls and patients receiving dialysis at 1.43 ng/mL and 1.09 ng/mL, respectively [75]. This evidence further demonstrates that acceptable concentrations of serum MK-7 can be quite high. Overall, VK, even the highly bioactive form of MK-7, is a safe supplement at relatively high daily doses. 

Some researchers believe that a Western diet contains an insufficient amount of VK, partly due to standard food manufacturing processes [26]. Moreover, in countries like the United Kingdom, the consumption of green leafy vegetables has reduced in recent years, causing a corresponding decline in VK intake [7]. Contrarily, it has been reported that people in other countries, like Japan, Germany, and the Netherlands, consume the recommended level of VK due to the differences in their typical diets [7]. In the United States, according to the 2011–2012 National Health and Nutrition Examination Survey, the mean dietary consumption of VK1 was above the recommended adequate intake levels in men and women over the age of 20 years [15]. As such, there is no consensus across the scientific literature regarding whether most people have a sufficient VK status or how to properly determine this beyond the observation of excessive bleeding [35]. Further research is clearly needed in this area. 

As with other nutrients, a person’s need for VK depends on age, sex, genetics, and other factors such as health status. For genetic contributions, various polymorphisms in the *APOE* gene, including E2, E3, and E4, have been identified; *APOE* codes for apolipoprotein E (ApoE), which is needed for VK transport through the body (Figure 2). However, the full effects of these polymorphisms on VK metabolism remain unknown due to conflicting study results [7,76]. Genetic variations in the *UBIAD1* gene have also been discovered [77]. These variants lead to a diminished conversion rate of VK1 to MK-4, causing the development of Schnyder corneal dystrophy, an opacification of the cornea due to excessive phospholipid and cholesterol deposits. Additionally, polymorphisms in vitamin K epoxide reductase complex subunit 1 (*VKORC1*) influence the inhibitory effects of warfarin (Figure 3), an anticoagulant treatment that prevents this enzyme from reducing VK [76,78]. Similarly, due to their role in the VK recycling pathway, *GGCX* and *VKORC1* have nucleotide polymorphisms that may alter the efficiency of Gla protein carboxylation [76]. Therefore, these genetic variations could also influence individual VK requirements. Lastly, *CYP4F2* codes for an enzyme that is partly responsible for the degradation and elimination of VK (Figure 2); the *CYP4F2* variant V433M is associated with the reduced hepatic excretion of VK due to its limited ability to initiate VK metabolism [76]. Hence, researchers suspect that carriers of the V433M allele may require less VK to meet their physiological requirements [76]. Moreover, *CYP4F2* alleles can alter the required dose of warfarin due to their effects on warfarin and VK metabolism [79]. Therefore, the genetic background has a role to play in affecting a person’s VK requirements due to its multifaceted influence on VK metabolism.

Various health-related factors can also contribute to VK requirements. The well-known health issues that alter VK requirements are cardiovascular problems, which are treated with VK antagonist anticoagulants. Examples of such conditions include atrial fibrillation, venous thromboembolism, and ischemic stroke [80]. Historically, many healthcare providers have recommended that patients on these VK antagonists refrain from consuming VK-rich foods due to their interference with the drug’s mechanism of action. However, a systematic review challenged this notion by demonstrating that dietary VK consumption is not a contraindication to VK antagonist treatment [80]. However, this study only looked at the effects of dietary VK on VK-dependent antagonists, not supplemental VK, which is in a more purified and highly bioactive form. Regarding this concern, Theuwissen et al. recommended against taking MK-7 supplements while on VK-dependent anticoagulant drug treatments such as acencoumarol, indandione, phenprocoumon, or warfarin because MK-7 can interfere with the mechanisms of action of these drugs [31]. Other diseases, including short bowel syndrome, celiac disease, cystic fibrosis, biliary atresia, and other gastrointestinal issues, predispose people to having higher VK needs than healthy individuals [7]. This is because conditions like these typically reduce the ability of VK to be absorbed across the gastrointestinal tract, thereby causing a heightened risk for VK deficiency [7]. Furthermore, the VK synthesis by the gut microbiota may also be negatively impaired in people with these diseases. Therefore, to prevent VK deficiencies, patients might need to be intentionally supplemented at a higher dose to overcome their predisposed VK absorptive and biosynthetic limitations. 

Age is also a relevant factor, but there is no consensus across the scientific literature regarding how age affects VK status. Some researchers argue that elderly people are more prone to VK deficiencies, while others say they are more resistant [7]. Differences in VK status observed in pre- and postmenopausal women have led researchers to hypothesize that estrogen also plays a role in VK metabolism [7]. However, until more research is carried out, the exact mechanism by which low levels of estrogen or age may impair VK metabolism will remain unknown. 

At the youngest end of the age spectrum, infants are particularly vulnerable to insufficient VK levels since VK does not cross the placenta, and breast milk concentrations are typically low [7]. This puts newborns at risk of developing hemorrhagic disease of the newborn (HDNB), which can be prevented either by administering 0.5–1 mg of VK1 intramuscularly or by providing an oral dose of 2.0 mg of VK1 [68,81]. However, this prophylactic use of VK1 is only beneficial for preventing HDNB for the first couple of weeks of life. After this time, breast milk or infant formula must contain sufficient VK levels to support proper coagulation [68]. VK1, MK-4, and MK-7 have all been detected in breast milk using high-performance liquid chromatography (HPLC) [82]. To raise the VK content of their milk, nursing mothers can supplement with VK, since a study showed that increasing maternal VK1 intake raises the MK-4 concentrations in breast milk [83]. Alternatively, commercial infant formula is enriched with 50–100 µg/L of VK1 to prevent VK deficiency.

## 6. Determination of Vitamin K Status

A variety of biomarkers can be used to assess individual VK status. However, some are more sensitive and specific than others. For example, serum VK1 concentrations can help determine VK status, and values below 0.15 µg/L indicate VK deficiency [39]. However, while the serum concentration of VK1 can be assessed within 24 h of consuming a VK-rich meal, there is much variation in the measured levels among and within individuals [7]. The detected level of VK1 is also closely linked to individual serum triglyceride levels, confounding the accuracy of its measurement [38]. Assuming VK is being sufficiently consumed, the higher the level of triglycerides in the blood, the higher the level of VK1 due to its lipophilic nature [7]. Therefore, a mathematical adjustment must be made to account for the serum lipid content when measuring blood VK1 concentrations [7]. Additionally, since VK1 is converted into MK-4 in many extrahepatic tissues, this measurement does not capture the total level of VK in the body [38]. Since this measurement only accounts for one type of dietary VK, it is unreliable for determining the total body VK concentration [7]. 

Several methods to directly quantify serum VK concentrations have been established. Examples of these methods include HPLC and liquid chromatography tandem mass spectrometry [73,84]. Electrochemical detection is also a sensitive and selective way to measure the concentrations of any form of VK [84]. Riphagen et al. outlined a method to quantify plasma VK1, MK-4, and MK-7 levels using HPLC tandem mass spectrometry [85]. However, one remaining challenge is the lack of scientific agreement on what a sufficient plasma VK concentration is [84]. Thus, further research is still required in this area.

Another option to evaluate a person’s VK status indirectly is by determining various coagulation times, including the internal normalized ratio (INR) and activated partial thromboplastin time [7]. However, this approach is impractical since the concentration of blood prothrombin must decrease to 50% of its normal physiological level before the INR detectably changes [7]. Additionally, other medical conditions involving the liver or blood can affect these measurements. This approach is, therefore, not sensitive enough for routine assessment and is not specific to VK [7]. 

Measuring the carboxylation status of various VK-dependent Gla proteins can also be used to assess VK status. Prothrombin is an excellent example of a Gla protein requiring VK activation. In its undercarboxylated form, it is called proteins induced in the VK absence or antagonism factor II (PIVKA-II) [7]. Measuring PIVKA-II levels can be a helpful indicator of the level of active prothrombin circulating in the bloodstream and is an indirect assessment of VK status. The relative concentration of carboxylated matrix Gla protein (cMGP) is also a biomarker of VK status. One group of researchers used a sandwich enzyme-linked immunosorbent assay to determine the plasma concentrations of cMGP and undercarboxylated matrix Gla protein (ucMGP); from this, the relative amount of cMGP was determined [86]. Likewise, the relative percentage of undercarboxylated osteocalcin available is another sensitive measurement that can contribute meaningfully to understanding someone’s VK status [7,73]. However, the undercarboxylated osteocalcin concentrations are affected by parathyroid hormone and VD status [84]. Moreover, this measurement is not correlated directly to a person’s blood VK1 concentration, confirming the necessity of using more than one biomarker to establish a person’s VK status [7]. 

Finally, VK metabolites excreted through the urine can be measured using HPLC to help determine a person’s overall VK status [38]. 5C-aglycone and 7C-aglycone, for example, are the two primary metabolites that increase and diminish correspondingly with VK1 and dihydrophylloquinone consumption [38]. The advantages of this analysis include its consideration of metabolites from all VK subtypes and noninvasive sampling method; the former benefit is especially relevant since none of the other aforementioned methods measure a metabolite common to all VK subtypes [38].

## 7. Impacts of Vitamin K on Human Health

### 7.1. Neurological Function and Disorders 

As previously mentioned, one essential role of VK2 is promoting brain health and function [29,87,88]. Interestingly, VK deficiencies are linked to a variety of neurodegenerative conditions like Parkinson’s disease and dementia [89,90]. Many neurological disorders like Alzheimer’s disease (AD), Parkinson’s disease, and brain ischemia result from oxidative damage and inflammation, which VK can help prevent [89,91]. For example, by inhibiting 12-LOX, VK shields premature oligodendrocytes from ROS damage, thus protecting against periventricular leukomalacia [43]. This defense mechanism is especially relevant in the developing infant brain, where inflammation, hypoxia, or ischemia can cause ROS accumulation [43]. Likewise, VK is necessary for the developing brains and nervous systems of children [92]. In seniors, VK’s antioxidant and neuroprotective benefits are also associated with a slower rate of cognitive decline [88,90,93,94]. Overall, for the young and elderly, VK has a variety of brain-health-promoting roles. 

VK has many mechanisms of action that support brain health. The discovery of VK’s role in promoting the biosynthesis of complex lipids necessary for optimal neurofunction began with Lev and Milford observing how treating *Bacteroides melaninogenicus* culture with VK1 initiated de novo synthesis of 3-ketodihydrosphingosine synthetase (3-KDS) [95]. The first enzymatic step in the sphingolipid biosynthetic pathway requires 3-KDS, and this step is common in *B. melaninogenicus* and yeast, as well as in the mammalian brain and liver [95]. 3-KDS is especially integral to the health of the central nervous system since this enzyme synthesizes sphingolipids like ceramides, sphingomyelin, cerebroside, sulfatide, and ganglioside, which are essential for cellular interactions, transformations, structures, myelin sheath formation, proliferation, senescence, and differentiation [93,96]. After Lev and Milford’s work, Sundaram and Lev bolstered these findings using the brains of warfarin-treated mice, showing a major reduction in sulfatides and minor reductions in cerebrosides and sphingomyelin [97]. These authors additionally concluded that the activity of sulfotransferase and arylsulfatase in the brain was enhanced by VK and inhibited by warfarin treatment. The mechanism by which VK accomplishes this enzymatic activation is thought to be through its γ-carboxylation activity or another post-translational modification [97]. Nevertheless, this action of VK in the brain is critical given that sulfotransferase and arylsulfatase are necessary to regulate sulfatide production for myelin sheath formation and brain development.

Another neuroprotective mechanism through which VK acts is by inhibiting amyloid β (Aβ)-induced cytotoxicity [91,98,99]. The toxic aggregation of these β strands is an important part of the neurodegenerative pathway leading to pathogenic conditions such as AD. However, in *Drosophila*, researchers showed that the levels of Aβ plaques reduced with the supplementation with VK2 [100]. One way through which VK2 promoted the clearance of these Aβ plaques was by upregulating the expression of autophagic proteins, such as Beclin 1 and LC3, to initiate the autophagy-lysosomal pathway [100]. VK also prevented Aβ plaque cytotoxicity by improving mitochondrial function. Because of this, the lifespans and climbing abilities of the *Drosophila* supplemented with VK2 increased, highlighting the beneficial effects of VK on cognition and health. Additionally, Huang et al. found that MK-4 treatment had a dose-dependent anticytotoxic effect on cells transfected with amyloid precursor protein [99]. These authors postulated that the primary means through which VK prevented Aβ-induced, caspase-3-mediated apoptosis was through activating phosphatidylinositol 3-kinase (PI3-K) associated protein kinase B and the Bcl-2-associated death promoter protein signaling pathway by the binding of the growth-arrest-specific gene 6 (Gas6) VK-dependent Gla protein to the Axl tyrosine kinase receptor. Moreover, they found that by inhibiting ROS formation, VK contributed to this prosurvival effect. Another inhibiting effect of VK2 on cytotoxically induced cell death of PC12 brain cells due to Aβ plaques is the attenuation of p38 MAPK pathway activation and cytochrome C release, as well as a reduced Bcl-associated X protein/Bcl-2 ratio [49]. Cumulatively, these studies demonstrate the multitude of pathways through which VK elicits neuroprotective mechanisms against Aβ-induced cytotoxicity.

Other studies have highlighted the effects of VK on the health of the aging brain. For example, Elkattawy and colleagues studied the effect of MK-7 on aged rat memory, behavior, social anxiety, levels of oxidative stress, cytokine production, hippocampal histology, and many other relevant parameters [29]. Interestingly, the aged rats treated with MK-7 (30 mg/kg once per day, five times per week for 17 months) outperformed the aged control rats on all social tests. Moreover, the treated aged rats did not have statistically significant differences in their social anxiety, social novelty, memory, or depressive behavior scores compared to the younger adult control group, highlighting the remarkable efficacy of VK2 for preventing age-related declines in normal social behavior. Biochemical measurements mirror this conclusion as well. For example, oxidative stress biomarkers decreased and antioxidant activity increased in the VK2 treatment group compared to those in the aged control group. Specifically, VK2 treatment effectively reduced the expression of the genes encoding IL-6 and TNF-α in the hippocampus and frontal cortex. As expected, higher expression levels of proinflammatory cytokines were found in the brains of the untreated aged rats, leading to oxidative stress, and neurodegeneration [29]. Overall, that study provides excellent evidence for the beneficial effects that MK-7 has on cognition and health, especially in the aging brain. 

There is also a strong relationship between VK and the amelioration of brain function, specifically regarding the role of the Gas6 VK-dependent Gla protein. In rats, Gas6 is synthesized in large amounts within the central nervous system throughout life; Gas6 contributes to cellular development, the prevention of apoptosis, chemotaxis, myelination, and mitogenesis [96]. Interestingly, the concentrations of Gas6 in the rat frontal cortex, striatum, and hippocampus decrease with age, implicating its role in the aging process [101]. Two signaling pathways elicited by Gas6 include PI3-K and MAPK, which help to prevent the degradation of gonadotropin-releasing hormone and promote the survival of hippocampal neurons, respectively, among other functions [96]. In addition to maintaining neuronal health, Gas6 protects glia and microglia from undergoing TNF-α-induced apoptosis and can attenuate the expression of other proinflammatory mediators [96]. A murine study expanded this finding by observing that Gas6 could enhance the microglia efferocytosis of apoptotic cells, lower the expression of inflammatory cytokines IL-6 and IL-1β, reduce blood–brain barrier (BBB) damage, and promote neurofunction after subarachnoid hemorrhage [102]. In this study, Gas6 reduced the extent of brain injury by activating the Axl receptor and Rho-family GTPases Rac family small GTPase 1 (Rac1) signaling pathways. Similarly, in patients with acute ischemic stroke, those with lower serum Axl concentrations had a higher risk for hemorrhagic transformation [103]. Upon genetic evaluations, patients with certain single-nucleotide polymorphisms in the *GAS6-AS1* gene also had a significant increase in their risk for hemorrhagic transformation, highlighting the importance of Gas6 in maintaining neurological homeostasis [103]. Overall, without the VK-dependent Gla carboxylation of Gas6, none of these neuroprotective functions would be able to occur, thus showing the necessity of VK in the maintenance of brain health.

Protein S is another VK-dependent Gla protein that plays a significant role in promoting brain health and modulating cellular signaling. Although it is mainly expressed in the liver and then released into the blood as an antithrombotic factor, protein S is also synthesized in the brain [104]. Here, it helps to prevent pathogenic coagulation during ischemic or hypoxic conditions [96]. For example, after a stroke, protein S reduced fibrin buildup, neuronal apoptosis, and neutrophil infiltration in a murine model [96]. Moreover, an in vitro study using primary human brain endothelial cells demonstrated that protein S protected cells from damage due to oxygen and glucose deprivation through binding to the Tyro3 tyrosine receptor and the sphingosine 1-phosphate receptor. Likewise, these researchers found that protein S could also limit ischemic and hypoxic damage to the BBB by Rac1-mediated cytoskeletal reorganization. Finally, research has demonstrated that the binding of protein S to Tyro3 enhanced the migration of neural stem/progenitor cells toward glioma cell apoptotic bodies and the subsequent phagocytosis of these fragments, which aided in suppressing brain tumor growth [105]. Overall, without the VK-dependent carboxylation of protein S, its functions would be impaired, thus demonstrating the importance of VK in promoting proper brain function.

Microbiome dysbiosis has significant implications for gut–brain axis communication and the pathogenesis of neurological diseases [106]. Therefore, enhancing microbiome health is critical for preventing and treating neurodegenerative diseases such as AD [91,106]. VK is a microbiome-modulating compound that has gained attention for improving gut barrier function and beneficial bacterial growth [107]. One in vitro study simulating the human intestinal microbial ecosystem demonstrated that the addition of VK1 increased the abundance of beneficial microbes, like *Faecalibacterium*, leading to the heightened production of short-chain fatty acids (SCFAs), namely, propionate, acetate, and butyrate, which possess anti-inflammatory, immunomodulatory, and neuroprotective properties, such as the protection of the BBB [106,107,108]. The attenuated production of beneficial SCFAs during microbiome dysbiosis impairs neurological function, as their binding to G-protein-coupled receptors in the central nervous system reduces. Additionally, their indirect antioxidant and anti-inflammatory benefits decreases [108]. This mechanism further supports VK’s usefulness for improving brain function. In another study investigating the impact of VK on the microbiota, mice received ampicillin to cause intestinal microbiota dysbiosis; however, the coadministration of VK2 helped attenuate this damaging antibiotic-induced effect [109]. When the antibiotic- and VK2-supplemented mice performed various cognitive assessment tests, including the elevated plus maze, passive avoidance, Morris water maze, and novel object recognition tests, they consistently surpassed the mice who only received the antibiotic treatment. This highlights an important role for VK in the gut–brain axis. Moreover, the synthesis of GSH in the brains of the mice subjected only to the antibiotic treatment was lower than in the mice administered VK2 plus antibiotics, indicating that the VK2-treated mice experienced less oxidative stress. In further support of this, VK2 administration considerably attenuated the levels of brain malonaldehyde synthesis and myeloid peroxidase activity, both biomarkers for neuroinflammation and oxidative stress. VK2 treatment also increased superoxide dismutase activity, a well-known antioxidant enzyme. At a morphological level, the intestinal villi height and crypt depth were shorter and shallower, respectively, in antibiotic-treated mice compared to mice administered VK2 plus antibiotics. This evidence illustrates how VK2 can also help protect against the antibiotic-associated impairment of gut barrier function [109]. Overall, these studies confirm the need for more research to better understand how VK supplementation can improve microbiome health to prevent age-related neurodegeneration. 

### 7.2. Cardiovascular Function and Disorders 

Many studies have linked calcium supplementation to increased mortality due to cardiovascular diseases, as calcium overwhelms VK-regulated blood calcium control mechanisms [26,110]. It has been well established that vascular calcification increases the risk of pathogenic blood coagulation partly due to the inflammation caused by atherosclerosis [26,111,112]. Specifically, in the absence of sufficient VK-dependent MGP carboxylation, calcified plaques build up in blood vessels, thereby increasing the thickness of the vascular wall [26]. This results in vascular stiffness and increased blood pressure [113], which contribute to cardiovascular diseases such as arterial thrombosis [111]. Therefore, VK deficiency is a risk factor for the development of atherosclerosis and cardiovascular injury [26,114,115].

Several studies delineate the relationship between VK status and pathogenic vascular calcification (calciphylaxis). Nigwekar et al. found that patients with a lower VK status, as seen in those with a higher relative ucMGP concentration, were also more likely to have calciphylaxis compared to the control group [86]. The percentage of VK-deficient patients in this study who contracted calciphylaxis was 90%, while those in the control group had a 50% case rate. In another study, patients with human immunodeficiency virus (HIV) with a VK deficiency, as indicated by high levels of dephosphorylated-ucMGP, also had higher coronary artery calcium scores that correlated to an increased risk for pathological cardiovascular conditions [116]. Furthermore, Luo et al. [117] demonstrated that in mice with a nonfunctional *Mgp* gene, arterial calcification and death occurred within the first two months of life due to the bursting of the abdominal or thoracic aorta. These authors proposed that MGP prevents calcification via its ability to bind minerals and ions. Scientists know now that MGP binds to both calcium and phosphorous ions, but MGP’s other mechanisms of action in limiting vascular calcification have also been discovered. These mechanisms include increasing osteocalcin production to redirect calcium to the bones and promoting macrophage phagocytic activity to clear apoptotic bodies [118]. Furthermore, a systematic review and meta-analysis demonstrated that increased plasma levels of ucMGP, an indicator of VK deficiency, were linked to heightened risks of all-cause mortality and cardiovascular disease events, while a reduced risk of coronary heart disease correlated with high levels of VK consumption [119]. On the other hand, another systematic review of controlled trials did not find any link between vascular calcification and VK supplementation [120]. Therefore, because of the limited studies available for analysis, researchers have called for further randomized clinical trials and prospective cohort studies to establish the role of VK more firmly in cardiovascular disease pathogenesis, as conclusive evidence has not been provided [119,120]. In general, there is moderate evidence supporting the critical role of the VK-dependent Gla protein MGP in preventing calcium-induced vascular injury. 

VD may also have a relevant role alongside VK in maintaining calcium homeostasis and in preventing cardiovascular injury [121,122]. Firstly, MGP, synthesized mainly in arterial vessel walls and cartilage, requires VD to enhance its expression, showing the synergistic functions of these two vitamins [123]. For example, one study demonstrated that supplementation with both VK1 and VD improved vascular elasticity compared to VD alone [123]. Another study assessed how VD and the level of undercarboxylated MGP, as an indicator of VK status, influenced blood pressure [113]. Study participants who had low 25-hydroxyvitamin D (≤50 nmol/L) and VK (dp-ucMGP ≥ 323 pmol/L) serum levels had the highest systolic and diastolic blood pressures. Moreover, in comparison to those with the highest VK and VD levels, the low-status group had a 69% greater risk of acquiring hypertension. Finally, a more recent study assessed the effect of combined VK and VD status on all-cause mortality and cardiovascular health in participants with a mean age of 70 [124]. Interestingly, the researchers found a significant increase in the left ventricular mass index, an indicator of poor cardiac structure, in the patients with the combined low VD and VK status compared to the other groups with an adequate status of one or both VD and VK. Although there were no other significant changes in the other echocardiographic measurements, the participants with the combined low VD and VK statuses still had a significantly higher rate of all-cause mortality [124]. Collectively, these studies highlight the importance of both VK and VD to ensure optimal cardiovascular health. However, further studies are warranted to delineate and confirm this complex relationship, as more evidence to support this conclusion is needed [121]. 

**Figure 4:**
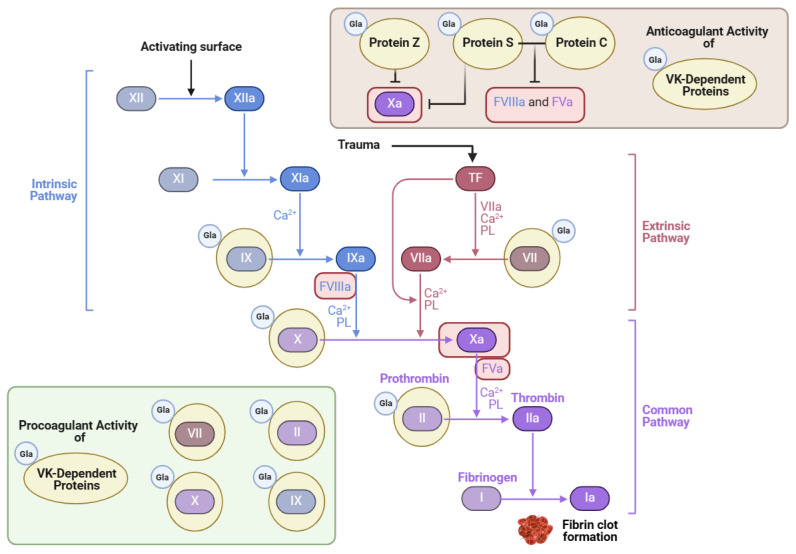
Vitamin K’s role in the coagulation cascade (adapted from “Coagulation Cascade”, created using BioRender.com (2024); retrieved from https://app.biorender.com/biorender-templates) accessed on 4 June 2024.

Thrombosis can be a complication of cardiovascular disease. Many patients who are at risk for various thrombotic disorders are treated with blood thinners. One notable class of blood thinners is 4-hydroxycoumarin derivatives, which include warfarin and acenocoumarol [125]. These drugs inhibit VK recycling by preventing VKORC1 from converting VK epoxide into VK, as depicted in Figure 3 [9]. The goal of antagonizing VKOR is to attenuate the activation of the hepatic clotting factors II, VII, IX, and X [9] (Figure 4). However, in doing so, the VK-dependent anticoagulant proteins are also inactivated. 

A few studies have investigated the dosing effects of MK-7 or VK1 for patients on anticoagulant therapy [9,36]. For example, Theuwissen et al. found that even 10 μg/day of MK-7 interfered with acencoumarol treatment [31]. In patients taking the MK-7 supplementation, the target INR decreased drastically below the desired value of 2.0, indicating that blood clotting was occurring too quickly. Consequently, patients receiving VK-targeted anticoagulant treatments such as acenocoumarol, indandione, phenprocoumon, or warfarin should not take any MK-7 supplements while on these blood thinners [31,78]. However, these authors did not note any contraindications for VK1 supplementation if the daily dose was below 100 μg/day. 

Although VK has a vital role in promoting blood coagulation, it also inhibits excessive levels of blood clot formation. Protein Z is one Gla protein with anticoagulant activity that blocks clotting factor Xa when bound to protein-Z-dependent protease inhibitor [4]. Protein C is also an anticoagulant Gla protein activated by protein S, its cofactor [126]. Together, these Gla proteins inactivate important coagulation factors like Va and VIIIa, as illustrated in Figure 4 [125,127]. In a blood analysis of patients with HIV, the activity of protein S decreased significantly in comparison to the control, which correlated with lower CD4^+^ T-cell counts [128]. Thrombotic events are widespread in patients with HIV and are attributed chiefly to the lack of protein S, which has an independent role in anticoagulation activity apart from protein C [128]. One example is its cofactor function with tissue factor (TF) pathway inhibitor (TFPI) [126]. Protein S bound to TFPI inhibits factor Xa, which blocks coagulation [127]. In mice lacking the gene encoding protein S, improper blood coagulation and abnormal vascular formation also ensued [127]. Therefore, VK’s role in the carboxylation of proteins S and C is essential for preventing excessive coagulation and promoting vascular homeostasis.

VK also maintains cardiovascular homeostasis via carboxylating Gas6 protein. This 75 kDa protein binds to Tyro3, Axl, and Mer (TAM) receptors, along with protein S. However, unlike protein S, Gas6 protein can bind to all three TAM receptors [4]. Through this TAM receptor binding, Gas6 increases the expression of TF to initiate the extrinsic coagulation pathway upon vascular injury; Gas6 also upregulates the expression of endothelial cell intercellular adhesion molecule-1 and vascular cell adhesion molecule-1 on endothelial cells, as well as P-selectin expression on both endothelial cells and platelets [127]. The result is platelet aggregation, leading to thrombogenesis, leukocyte sequestration, and inflammation, highlighting the importance of Gas6 in forming thrombi and regulating the immune response during blood vessel damage [127]. Another study corroborated these findings by showing that *Gas6* knockout mice had lower thrombosis levels and tissue factor activity [127]. Another human study provided additional support by showing that higher Gas6 levels were linked to venous thrombosis [129]. However, other studies demonstrated that intravenous VK1 treatment only marginally increases overall Gas6 blood concentrations, and future research must test VK’s effect on Gas6’s carboxylation status in vivo [130]. Altogether, Gas6 is a critical Gla protein with pertinent cardiovascular functions, demonstrating the necessity of VK in this system.

### 7.3. Kidney Function and Disorders

The kidney is another organ involved in VK metabolism and storage. The renal reserves of VK are mainly in the form of MK-4 [131]. However, the metabolic activity of VK in the kidney, and throughout the body, is prone to dysregulation when renal pathologies arise. For example, during the development of rat chronic kidney disease (CKD), renal MK-4 levels rose, but *UBIAD1* and *VKORC1* expression decreased [131]. VK recycling is also systemically impaired due to uremia, a condition often resulting from CKD [132]. In rats with uremia induced through dietary adenine supplementation, pathogenic calcification in the vasculature and kidney arose due to low levels of VK-dependent cMGP; however, high levels of VK2 or VK1 supplementation were able to rescue rats from this detrimental effect by reducing heart and kidney calcification and substantially increasing GGCX activity in the liver [133]. Another study confirmed this connection by finding that a low VK status in a CKD rodent model predisposed the rats to increased levels of vasculature calcification both in the kidney and throughout the body [134]. Compared to healthy controls, people with CKD also have lower levels of MGP, along with significantly reduced incorporation of MK-7 into HDLs [135]. Interestingly, that study noted that in contrast to MK-7, MK-4 was packaged in LDLs and HDLs more effectively in the uremic group of patients with CKD on hemodialysis than in the healthy control group, highlighting the changes in VK metabolism and distribution during CKD. Nevertheless, what remains to be determined is if lowered and altered VK activity precedes kidney disease or if it is a consequence of kidney disease.

These decreased VK levels seen in patients with CKD often increase the risk of other health complications [131,136,137]. One negative health outcome that they are prone to developing is cardiovascular disease. Without the VK-dependent activation of MGP, calcium is excessively deposited in blood vessels, which leads to vascular stiffness, atherosclerotic plaques and cardiovascular injury [137,138]. Additionally, patients with CKD eventually show other signs of mineral and bone disorders linked to improper calcium homeostasis, including a heightened risk for bone fractures due to high levels of undercarboxylated osteocalcin [132]. This is because, in addition to MGP, other Gla proteins throughout the body such as osteocalcin become undercarboxylated, and PIVKA-II levels also increase in patients with CKD [139]. However, VK-deficient patients on hemodialysis ameliorated their VK status through VK2 supplementation [140]. Thus, there is emerging evidence that supplementing patients with CKD with VK may yield some protective health benefits. 

In addition to the dysregulated metabolism of VK seen in people with kidney issues, corresponding medical treatments, conditions, and recommendations often unintentionally lead to reduced VK status. For example, the CKD dietary restrictions of potassium- and phosphate-rich foods typically lead to a concomitant decline in VK intake [132,138]. Additionally, prescribing certain phosphate-binding drugs to patients with CKD can reduce VK2 levels through their additional ability to bind VK2 [132]. Some researchers also suggested that the subsequent dysregulation of the microbiome or gastrointestinal tract may contribute to the low VK status of patients with kidney diseases [132]. Due to these factors, patients with CKD are at a heightened risk for developing VK deficiencies and may benefit from VK supplementation. 

### 7.4. Bone Function and Disorders 

VK also plays an essential role in supporting bone health and preventing bone pathologies. Various studies have found that a low VK status is associated with osteoporosis, osteopenia, poor bone formation, elevated fracture risk, and low bone mineral density [26,69,141]. Many of these harmful outcomes are attributed to low levels of carboxylated Gla proteins such as MGP and osteocalcin [26,141,142]. Moreover, researchers reviewing the effects of VK antagonist anticoagulant treatments have found that these drugs also detrimentally impacted bone metabolism [143]. Specifically, they reported that a deficit of VK-dependent osteocalcin carboxylation in those taking VK antagonist drugs led to poor hydroxyapatite formation in the bone matrix and a higher risk of osteoporosis [143]. Overall, VK-dependent carboxylation of Gla proteins critically affects bone health and warrants further investigation to determine VK’s potential therapeutic role in preventing bone pathologies. 

MGP is one relevant Gla protein that prevents many bone-related diseases and pathological mineralization in chondrocytes [4,117]. Luo et al. found that undesired and disorganized cartilage calcification occurred in mice with mutant *MGP* genes, leading to the improper formation of the growth plate chondrocyte columns, which are needed for adequate bone matrix deposition and sufficient growth [117]. Consequently, these mutant mice were shorter and did not grow as quickly as their wild-type counterparts. Additionally, the mutant mice incurred osteopenia and fractures, showing how MGP promotes necessary bone growth and density. 

VK-dependent GGCX carboxylates another relevant Gla protein called Gla-rich protein (GRP), which prevents undesired calcification [4]. Although its exact mechanism of action is not understood completely, GRP prevents soft tissue calcification and regulates calcium availability [144,145]. Specifically, carboxylated GRP induces the expression of the gene encoding α-smooth muscle actin and decreases osteopontin protein expression to reduce undesired mineralization [145]. Another study involving zebrafish showed the necessity of GRP for proper skeletal growth and development; specifically, when the gene encoding GRP was knocked out, total fish length shortened, and notochord development was disrupted [146]. Since the gene encoding GRP is highly conserved between zebrafish and other vertebrates, this also implicates GRP in human skeletal formation. Moreover, both carboxylated GRP (cGRP) and undercarboxylated GRP (ucGRP) elicit anti-inflammatory effects on chondrocytes and synoviocytes in vitro [144]. In this study, GRP reduced inflammation by attenuating the accumulation of prostaglandin E2 and expression of the genes encoding cyclooxygenase-2 and matrix metalloprotein 13. Therefore, VK-dependent cGRP may help hinder the pathogenesis of debilitating inflammatory conditions like osteoarthritis.

Another function of VK is the carboxylation of the osteocalcin Gla protein, which is synthesized by osteoblasts and sequesters calcium within the hydroxyapatite of the inner matrix of the bone [26]. Interestingly, VD is also required to activate osteocalcin, and studies have revealed a VD-receptor-response element within the promoter of the gene encoding osteocalcin (*BGLAP*) [147]. Thus, VD initiates the transcription of *BGLAP* before VK carboxylates it [148]. As evidence of these critical functions, several studies have demonstrated that the risk for bone fractures increases when the plasma concentrations of VK and VD are low [147]. A recent meta-analysis of randomized controlled trials also affirmed this, validating the roles of both VK and VD in improving bone mineral density and carboxylated osteocalcin levels [149]. VK and VD supplementation can even increase bone mineral density to lower the risk of postmenopausal osteoporosis in women, and VD enhances bone strength alongside VK by promoting intestinal calcium uptake [147]. Thus, many studies have highlighted the synergistic benefits of VD and VK on bone health.

Several cytokines, however, antagonize the roles of VK and VD in maintaining strong bones and optimal levels of osteocalcin. TNF-α, for example, prevents the transcriptional activation of *BGLAP* at the level of the VDR and can inhibit osteoblast differentiation and migration [148,150]. TNF-α also activates the transcription of the gene encoding NF-*κ*B, whose p65 subunit prevents steroid coactivator-1 from binding to the VDR, thereby inhibiting VD-mediated transcriptional activity [148]. Moreover, NF-*κ*B suppresses osteoblast development and increases osteoclast differentiation, which can lead to bone resorption [150]. Interestingly, supplementation with VK can overcome the antagonistic effects of proinflammatory cytokines to maintain bone health. For example, MK-7, but not VK1, can effectively suppress both NF-*κ*B and TNF-α activities via a GGCX-independent mechanism [150]. Additionally, VK2 can lower the protein expression of the NF-*κ*B ligand receptor (RANKL), which is responsible for osteoclast function and development [151]. In patients with rheumatoid arthritis supplemented with VK2, less bone resorption occurred due to attenuated levels of RANKL [151]. Overall, because of these protective effects and enhancement in VD activity, VK2 possesses therapeutic potential to combat rheumatoid arthritis, bone loss, osteoarthritis, and other bone disorders. VK2 is currently used to treat osteoarthritis in Japan [150].

In addition to the carboxylation of relevant Gla proteins and the role of VK in regulating cytokine activity, VK has one additional known mechanism of action involved in maintaining bone homeostasis. This mechanism involves VK binding to SXR to form a heterodimer with RXR, which then acts as a transcription factor for the genes encoding anabolic osteoblast proteins alkaline phosphatase, osteopontin, MGP, and osteoprotegerin after binding to the SXRE [52]. Using human osteoblast cell lines, Azuma et al. found that VK also promoted the expression of many other genes through SXR-dependent pathways, including those encoding CD14, a pattern-recognition receptor that regulates osteoblastogenesis and osteoclastogenesis; tsukushi, a driver of collagen accumulation; and matrillain-2, found in the extracellular matrix [53]. Thus, the authors concluded that VK may promote bone deposition through SXR-binding activity and have therapeutic effects on treating bone disorders. 

### 7.5. Immunomodulation and Immune Disorders 

VK has an essential role in supporting immune system function and preventing infectious diseases. In particular, some VK-dependent Gla proteins contribute to immune system regulation [4]. For example, protein S helps to maintain homeostasis within the vascular and immune systems [4]. Approximately 60% of protein S is bound to the β chain of the regulatory C4b-binding protein (C4BP), and it is postulated that this allows C4BP to bind to negatively charged phospholipid membranes, facilitating complement regulatory activity [126]. Additionally, both protein S and Gas6 can individually bind to the Tyro3 and Mer TAM receptors found on phagocytic cells like dendritic cells and macrophages; this facilitates the efferocytosis of apoptotic cells, thereby preventing the excessive inflammation induced by the innate immune response [126]. TAM receptor binding also attenuated LPS-stimulated proinflammatory cytokine production by antigen-presenting cells [127]. Moreover, when Gas6 binds to Mer, renal inflammation is reduced during glomerulonephritis, possibly due to the lower expression of NF-κB [127]. However, renal inflammation is enhanced when Gas6 binds to the Axl TAM receptor, which initiates mesangial cell proliferation. Overall, both Gas6 and protein S elicit anti-inflammatory and some inflammatory pathways, revealing complex mechanisms of VK-dependent immunomodulation. 

For certain infectious diseases like coronavirus disease 2019 (COVID-19), having a sufficient VK status may limit the development of severe symptoms. In blood samples of patients with COVID-19, researchers found higher levels of undercarboxylated dephosphorylated MGP, indicating lower VK status compared to the healthy control patients [152]. Since VK has demonstrated protective effects against various lung pathologies, inflammatory cytokine release, the production of the acute-phase C-reactive protein, and other health issues, researchers proposed that this may be why low VK status is associated with severe COVID-19 outcomes [152]. Additionally, insufficient VK status may enhance ferroptosis, resulting in increased viral replication, inflammation, and cellular damage [153]. Overall, low levels of VK and VD are risk factors for severe COVID-19 [152,154,155], implicating both these vitamins in supporting resistance to infection. VK binding to the severe acute respiratory syndrome coronavirus 2 (SARS-CoV-2) spike protein (SP) may be another way that VK limits severe COVID-19. Researchers used the Bristol University Docking Engine molecular simulation to assess SP ligand binding in silico, focusing on a newly discovered fatty acid binding pocket within SP [156]. Interestingly, of all the tested compounds, VK2 had the highest binding affinity. Notably, VK1 ranked 70th among the tested ligands, indicating the relevance of the structural differences between VK1 and VK2. Additionally, a previous study confirmed that all coronaviruses known to infect humans have this fatty-acid-binding pocket [157]. Therefore, it is plausible that VK could also offer protection against SARS-CoV-2 and other coronaviruses. It is hypothesized that the potential protection provided by VK is due to its induction of the closed conformational state of SP, thereby preventing effective SP binding to its target ACE2 receptor [156]. However, although this research looks promising, further studies are warranted to validate these predictions. 

VK also possesses several other properties that may reduce the incidence or severity of COVID-19. Since ferroptosis is a pathological consequence of COVID-19, VK’s antioxidant and anti-inflammatory mechanisms may be valuable in helping prevent COVID-19-related ferroptosis [153]. Moreover, having a sufficient VK status also reduces typical lung pathologies in patients with COVID-19, such as pulmonary thromboembolisms and other acute lung injuries, in part due to the VK-dependent carboxylation of pulmonary MGP, which inhibits pathogenic lung calcification [153,158]. An association between SARS-CoV-2 infection and an altered microbiome has also been previously reported, which could impact the host’s immune response [159,160]. Therefore, VK’s positive impact on the gut microbiota may help to prevent immediate COVID-19-related microbial dysbiosis [161]. Even after mild cases, SARS-CoV-2 infections may lead to long-term destabilized gut microbiota [162], showing the necessity of supplementing with microbiome-enhancing nutrients, such as VK. Overall, VK has many properties supporting its usefulness in combatting COVID-19.

Gut microbiota dysbiosis can contribute to a variety of other diseases, one of which is type 2 diabetes mellitus (T2DM) [163]. However, through microbiota modulation, VK2 can improve glucose metabolism and insulin sensitivity. In comparison to a nonsupplemented control group, when patients with T2DM were supplemented with MK-7 for 6 months, the ratio of *Firmicutes* to *Bacteroidetes* was significantly reduced, the microbiota population density and diversity was enhanced, and the microbial production of SCFAs and secondary bile acids (SBAs) increased [163]. MK-7 also decreased the rate of decline of the other bacterial species observed in the patients supplemented with T2DM [163]. Interestingly, select metabolites and bacterial genera that were increased in the fecal samples of those supplemented with MK-7 were linked to reduced fat mass, total cholesterol, blood pressure, and hip circumference [163]. Moreover, the observed enhanced production of SCFAs and SBAs is known to improve the host immune response due to their ability to regulate inflammatory cytokines, T cells, and B cells, as well as support gut barrier function. Due to these beneficial effects of the gut microbiota populations and their metabolites, the fasting serum glucose and insulin levels were lower in VK-supplemented patients with T2DM compared to those in the nonsupplemented control group, demonstrating an improvement in glucose tolerance and insulin sensitivity. Moreover, both VK1 and VK2 intakes are associated with lower risk for T2DM [164]. Therefore, VK, especially VK2, has many therapeutic properties that may be utilized to treat T2DM through gut microbiome modulation. 

VK’s alteration of gastrointestinal microbial populations also has a significant effect on a person’s resilience against other diseases. Rheumatoid arthritis, as an example, is linked to microbiota dysbiosis [165]. Like with T2DM, researchers have concluded that VK may promote immune function and microbiota health in patients with rheumatoid arthritis to protect bone and joint quality [165]. Inflammatory bowel disease is another notable gastrointestinal issue linked to both VK and VD deficiencies [166]. One review attributed the ability of VK to reduce oxidative damage by scavenging free radicals, increase GSH activity, and suppress inflammatory signaling pathways to how it helps to mitigate tissue damage due to inflammatory bowel disease [161]. This review also highlighted the improvement in SCFA synthesis that VK modulates as another beneficial VK-dependent immune-enhancing effect. Therefore, VK supplementation may be valuable in patients with debilitating conditions by reducing inflammation and increasing gut barrier function through its microbiome modulatory properties.

Atopic skin disease is another immune-mediated condition that VK may be helpful in treating or preventing. For example, VK has been successfully used to suppress the proliferation of T-cell mitogen-activated peripheral blood mononuclear cells originating from pediatric patients with atopic dermatitis [167]. The subsequent cytokine production by these cells also decreased, and the T-cell functions were beneficially regulated when treated with VK, showing the potential therapeutic effects of VK. Since atopic dermatitis is a chronic inflammatory condition often requiring immunosuppressive treatments with harmful side effects, finding alternatives like VK may be favorable for certain patients [167]. Another study confirmed this finding, demonstrating that VK2 dose-dependently immunosuppressed peripheral blood mononuclear cells from pediatric with atopic dermatitis [168]. Overall, VK has presented a variety of beneficial effects in promoting the proper regulation of immune system function, thus possibly aiding in the prevention and treatment of many different diseases. 

## 8. Impacts of VK on Domestic Animal Health 

VK is not only significant to human health but also to animal health. For example, dogs can be susceptible to VK deficiencies when they have poor nutrient absorption from the gastrointestinal tract, have low intestinal bacterial VK biosynthesis, consume VK-antagonist rodenticides, or suffer concurrent health issues [169]. The most common occurrence is during anticoagulant rodenticide toxicosis. The VK antagonists found in rodenticides include warfarin and indandione derivatives [169]. When ingested by canids, these drugs inhibit VK recycling, thus preventing the activation of necessary clotting factors, which can subsequently cause internal and external hemorrhaging [170]. One treatment for this condition is the intravenous administration of VK1, which effectively reestablishes blood clotting homeostasis [170,171]. Although there have not been any clinical VK deficiencies reported in normal dogs, the National Research Council still includes a section for canine VK requirements in their Nutrient Requirements of Dogs and Cats (2006). However, the form of VK added to pet food is different from naturally occurring VK1 or VK2 in human food due to the damaging effects of the extrusion, drying, and enrobing processes. Instead, a VK3 derivative, such as menadione sodium bisulfite, menadione dimethylpyrimidinol bisulfite, or menadione nicotinamide bisulfite, is recommended [169]. Adult dogs should receive at least 22 µg menadione per kg of body weight per day, and puppies should consume at least 44 µg menadione per kg of body weight per day [169]. As in humans, there is little evidence or research regarding the toxic dose of naturally occurring VK in dogs, so the risk of hypervitaminosis appears to be of no major concern. The only reports of toxicity were due to the parenteral administration of VK3 or extremely high levels of dietary VK3 [169].

Cats can also develop VK deficiencies under rare circumstances. In feeding trials by the American Association of Feed Control Officials, queens and kittens that consumed canned food composed mainly of tuna or salmon developed VK deficiency [172]. In these trials, many of the cats suffered internal hemorrhage, elevated coagulation time, and even death. However, the surviving queens returned to normal health after therapeutic VK1 administration. Subsequent feline dietary trials attempting to reproduce VK-deficient signs using a low VK content and known VK-antagonistic compounds were unsuccessful, leading researchers to conclude that there must have been unknown anti-VK factors in the seafood-rich diets [172]. Since many feline diets are seafood-based and may cause VK deficiency, the NRC recommends that 1.0 mg menadione per kg be added to feline diets [169]. No recommendation for VK supplementation in other nonfish feline dietary types exists since it is assumed that gut microbial synthesis meets VK requirements [169]. Another way for cats to develop VK deficiency is through the consumption of dicumarol rodenticides. They also can acquire secondary VK deficiencies due to malabsorptive issues caused by enteritis, inflammatory bowel disease, cholangiohepatitis, hepatic lymphoma, or hepatic lipidosis [173]. In these cases, VK treatment is warranted and has shown promising results by reinstating PIVKA clotting times to homeostatic levels [173]. As with dogs and humans, no toxic VK dose has been reported, other than when administering VK3 parenterally or when extremely high dietary levels of VK3 are consumed [169].

Farm animals also require sufficient VK levels to promote overall health and prevent coagulopathies. For instance, the first acknowledgment of VK occurred after scientists noted that an unknown lipid-soluble vitamin prevented hemorrhaging in chicks [1]. Now, dietary VK3 supplementation is known to improve bone volume and formation in chickens [174]. Furthermore, chickens supplemented with enhanced levels of VK3 laid higher-quality eggs; this treatment led to an increased eggshell thickness and weight, egg yolk color, and egg MK-4 content [175]. 

Additionally, to avoid improper blood clotting in pigs, the NRC has instituted a dietary requirement of 0.5 mg of VK per kg feed across all production stages [176]. VK supplementation is especially critical for pigs consuming mold-contaminated feed ingredients, as certain mycotoxins can interfere with intestinal VK biosynthesis and normal blood clotting processes [176]. Researchers also hypothesized that changes in the porcine microbiota caused by antibiotic administration or antinutritional factors could alter intestinal VK synthesis, thereby impacting overall pig health [177]. 

In cattle, VK1 injections have been used to treat hemorrhagic diathesis and other bleeding disorders [178]. Dicoumarol toxicosis due to the ingestion of sweet moldy clover is one such hemorrhagic condition that VK1 helps to resolve [179]. In healthy cows, intramuscular VK1 injections were used to increase serum platelet counts and caused no adverse effects, showing the safety of such a treatment [178]. However, under normal circumstances, the biosynthesis of VK by ruminal and intestinal bacteria is likely sufficient to meet daily requirements [178]. Researchers have discovered that VK is even stored in bovine skeletal muscle tissues, indicating that VK may also affect muscle function [180]. Additionally, VK3 supplementation to dairy cows increased plasma and milk MK-4 levels, as well as propionate production by ruminal *Propionibacterium*, showing how VK supplementation impacts many aspects of dairy cow physiology [181]. Another promising study demonstrated how bovine endometrial epithelial cell treatment with MK-4 regulated the expression of genes required for successful reproductive events, including those coding for cytokines, growth factors, and proteins involved in the MAPK cascade signaling pathway [182]. In vitro MK-4 treatment of bovine peripheral blood mononuclear cells also stimulated cellular proliferation, showing a possible way for VK to modulate the bovine immune system [183]. Although more research is necessary in this area, VK has thus far been found to promote the optimal health and productivity of farms and companion animals. 

## 9. Conclusions 

In conclusion, VK is a valuable biomolecule with roles in human and animal health beyond its well-known function in blood coagulation, including neuroprotection, prevention of cardiovascular diseases, bone maintenance, and immune system regulation. As such, VK may have therapeutic benefits to reduce oxidative stress, gut microbial dysbiosis, T2DM development, pathogenic thrombosis, and much more. Due to its multifaceted beneficial properties, more research is warranted to elucidate further its mechanisms of action and how much should be consumed appropriately to support overall health.

## Figures and Tables

**Table 1:** The functions of selected vitamin-K-dependent Gla proteins covered in this review.

Gla Protein	Functions
Osteocalcin	Sequestering calcium in bone hydroxyapatite. Promotion of bone health and strength.
Matrix Gla protein (MGP)	Binding calcium to prevent undesired calcification in cartilage and vasculature. Promotion of proper bone growth and density.
Gla-rich proteins (GRPs)	Prevention of soft tissue calcification and regulation of calcium availability. Anti-inflammatory effects.
Growth arrest-specific gene 6 (Gas6)	Apoptosis prevention. Neuroprotection and central nervous system regulation. Prothrombotic activity. Regulation of immune system activity and inflammation.
Clotting factors II, VII, IX, X	Essential activity in the coagulation cascade.
Protein S	Inhibits FVIIIa and FVa in the coagulation cascade. Cofactor inhibition of clotting factor Xa. Promotes brain health by immunomodulation, cellular signaling regulation, and reducing oxidative damage. Modulation of immune system cells and inflammation.
Protein C	Inhibits FVIIIa and FVa in the coagulation cascade.
Protein Z	Cofactor inhibition of clotting factor Xa

**Table 2:** Recommended vitamin K intake for population cohorts by age and sex from the American Institute of Medicine [68].

**Infants**	**µg/day**
0–6 months	2.0
7–12 months	2.5
**Children**	**µg/day**
1–3 years	30
4–8 years	55
**Men**	**µg/day**
9–13 years	60
14–18 years	75
19–70+	120
**Women**	**µg/day**
9–13 years	60
14–18 years	75
19–70+	90
